# Identification of novel keloid biomarkers through Profiling of Tissue Biopsies versus Cell Cultures in Keloid Margin specimens Compared to adjacent Normal Skin

**Published:** 2010-04-07

**Authors:** Barbara Shih, Duncan Angus McGrouther, Ardeshir Bayat

**Affiliations:** ^a^Plastic & Reconstructive Surgery Research, Epithelial Sciences, School of Translational Medicine, Manchester Interdisciplinary Biocentre, University of Manchester, Manchester, United Kingdom; ^b^Manchester Academic Health Science Centre, Department of Plastic and Reconstructive Surgery, University Hospital of South Manchester NHS Foundtion, Wythenshawe Hospital, Southmoor Road, Manchester, United Kingdom

## Abstract

**Objective:** Keloid disease (KD) is a benign fibroproliferative skin tumor that results from abnormal wound healing and has no single definitive treatment. This study aims to identify KD biomarkers, which are cellular mediators that can serve as indicators of normal, pathological, and therapeutic processes. **Methods:** Bioinformatics analytic approaches, including comprehensive literature searches and DAVID Bioinformatics Resources 2008, were performed on the established KD linkage and previously reported microarray data to identify potential candidate genes for the study. Keloid margins and unaffected skin were obtained from KD patients (*n* = 4). RNA was extracted from the biopsies and second-passage culture equivalents. Reverse-transcriptase quantitative polymerase chain reactions were used to determine the gene expression levels. Student *t* tests were used to analyze the statistical significance in differential gene expressions. **Results:** Nineteen candidate genes were initially selected by bioinformatics analysis. Of the 19 genes, 10 were significantly (*P* < .05) upregulated in keloid margin biopsy specimens. The top-5 fold changes range from 10-fold to 175-fold, including aggrecan; asporin; inhibin, beta A; tumor necrosis factor-α inducible protein 6; and chromosome 5 open reading frame 13. There was no significant differential gene expression between the fibroblasts established using keloid margin or internal control sites. **Conclusions:** The transcriptomic data generated from cultures did not consistently correlate to the biopsy equivalents. This study has demonstrated 10 genes that are significantly upregulated in biopsy samples of keloid margin, 5 of which have a fold change higher than 10-fold. Importantly these genes may serve as a potential biomarker for KD.

Keloid disease (KD) is a benign dermal fibroproliferative tumor unique to humans which is thought to occur following an abnormal wound healing process.^1^ Keloid scars are aesthetically disfiguring often impair function as they can restrict skin and joint mobility, and have the potential to cause intense symptomatic (itch and pain) distress.^2^ There is currently no satisfactory treatment of KD because high recurrence rates and undesirable side effects have been observed irrespective of the intervention.^3^

Hence, the establishment of more effective therapeutic strategies, better understanding and characterization of the molecular mechanisms involved in KD are considered important developments. Biomarkers are biological mediators that may be used as an indicator of normal biological processes, pathogenic mechanisms or pharmacologic responses to a therapeutic intervention.^4^ By identifying new KD biomarkers, the disease process and treatment approach may be better characterized.

In contrast to KD, hypertrophic scars, another form of excessive raised dermal scarring, rarely reoccur after excision.^5^ Unlike hypertrophic scars, KD characteristically extends beyond the original wound boundary^6^. The margin of KD spreads into the surrounding healthy skin through invasion, rather than expansion, with a leading edge that is often erythematous and pruitic.^6^,^7^ The unusual invasive properties of the KD margin make it an interesting target to study and compare with normal skin.

When keloid-derived fibroblasts, the major cell type in KD, are compared with fibroblasts derived from normal skin or hypertrophic scars, the keloid-derived fibroblasts show several abnormal changes including excessive extracellular matrix production and proliferation, altered apoptosis, growth factor response and cytokine production.^3^ Although the use of tissue cultures would allow studying the gene expressions of a single cell type, such as a fibroblast, researchers often neglected the changes in cellular environment culturing conditions introduce. There are currently a limited number of studies that have examined gene expression levels in biopsy and cell culture samples simultaneously.^8^^-^11 In this study, we compared gene expression levels in tissue biopsy and cell culture, which were both derived from the same biological sample obtained from the same individual and then compared with similar samples harvested from different individuals.

This study, using bioinformatics analysis, aims to select the most freqently reported genes from previous literature of keloid susceptibility loci and existing microarray data by the frequency they have been reported. The gene expression levels in the margins of KD specimens are compared with those of unaffected skin from the same patient. Additionally gene expression levels in tissue biopsies will also be compared with those of tissue cultures to determine whether similar results are observed.

## MATERIAL AND METHODS

### Patients and samples

**Figure 1:**
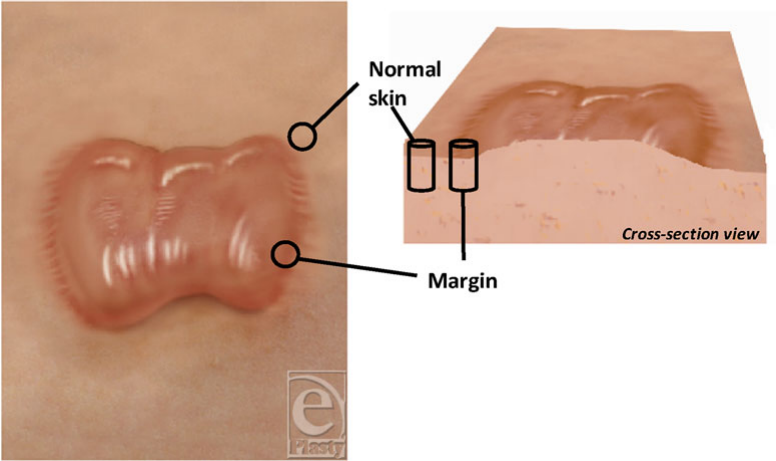
Illustration of the lesional sites of keloids taken in this study.

Samples from 4 patients were used in this study. The mean age was 29 ± 4 years. Three patients were white, and the fourth patient was of white/black Caribbean ancestry (Table 1). Biopsies of normal skin and keloids margin were obtained (Fig 1).

### Tissue culture

Primary tissue cultures were obtained by enzymatic digestion of biopsies. The collected samples were minced into small pieces with sterile scalpels and incubated in 0.25% to 5% collagenase A solution (Roche Diagnostics GmbH, Mannheim, Germany) at 37°C for 2.5 to 3 hours. The collagenase digestion was inhibited using fibroblast culturing media. The fibroblast culturing media consists of Dulbecco's modified Eagle's medium 3 (Lonza, Verviers, Belgium), supplemented with 10% heat-inactivated fetal bovine serum (Sigma, Gillingham, UK), 1% penicillin/streptomycin (Lonza), and 1% non–essential amino acids (Lonza).

After the digestion, each sample was spun at 1,200 rpm for 5 minutes, re-suspended in fibroblast culturing media, and seeded in 25-cm^2^ culturing flask (Corning, Corning, NY). The cultures were maintained at 37°C and 5% CO_2_, and the media was replaced every 48 hours. Passaging was carried out with trypsin-ethylene diamine tetraacetic acid (200 mg/L of ethylene diamine tetraacetic acid, 500 mg/L of trypsin; Lonza) when approximately 80% confluence was reached.

### RNA extraction

Four pieces of 2-mm^3^ tissue were cut off from each biopsy sample that had been stored in RNAlater. Each piece of 2-mm^3^ tissue was finely diced and placed in 2-mL round-bottomed Eppendorf tubes with a flame-sterilized steel ball bearing and 1 mL of Trizol (Invitrogen, Carlsbad, Calif). The tissues in the Epppendorf tubes were homogenized by Qiagen tissue lyser (Qiagen, Hilden, Germany) that was set at 30 beats per second for 12 minutes. The homogenized tissue suspension was transferred to a sterile 1.5-mL Eppendorf tube and centrifuged at 13,000 rpm for 10 minutes to remove cell debris. After the centrifugation, the resulting supernatant were transferred to a new Eppendorf tube and mixed with 0.2 mL of chloroform/1 mL of Trizol (Invitrogen). Solutions in each tube were mixed well and left at room temperature for 2 minutes, after which the mixtures were centrifuged at 13,000 rpm for 15 minutes. The upper aqueous layer in each tube was transferred into a fresh Eppendorf tube and equal volume of 70% ethanol was added and mixed well.

The extracted RNA was further processed using RNeasy kit (Qiagen, UK) according the manufacturer's instructions, followed by DNase treatment with a DNAFree kit (Ambion, Austin, Tex) according to the manufacturer's protocol.

### Complementary DNA synthesis

SuperScript II reverse transcriptase kit (Invitrogen) was used for synthesis of complementary DNA (cDNA). For each sample, 1000 ng of RNA, 1 µL of nucleotides mix (10 mM for each nucleotide) (Invitrogen), 375 ng of oligo-dT, 62.5 ng of random primers, and sterile nuclease-free water (Ambion) were mixed in a nuclease-free Eppendorf tube to make up a total volume of 12 µL. After incubation at 65°C for 5 minutes and rapid cooling on ice, 2 µL of 0.1 M of DTT (Invitrogen), 1 µL of RNaseOut (Invitrogen), and 4 µL of first-strand buffer (250 mM of Tris-hydrochloride, pH 8.3 at room temperature; 375 mM of potassium chloride; 15 mM of magnesium chloride; Invitrogen) were added to each tube. After incubation at 25°C for 2 minutes, 1 µL of SuperScript II reverse transcriptase (Invitrogen) was added and incubated at 25°C for a further 10 minutes, before being transferred to 42°C incubation for 50 minutes. Following this, the samples are incubated at 70°C for 10 minutes to inactivate the enzymes.

### Selection for candidate genes

Bioinformatics methods were used to select candidate genes. Through literature searches, 7 non–pathway-specific microarray gene profiling studies were identified, including studies of Smith et al,^12^ Seifert et al,^7^ Hu et al,^13^ Naitoh et al,^14^ Satish et al,^15^ and Chen et al,^16^,^17^ and a list of genes from own unpublished data (Table 2). Full list of upregulated or downregulated genes were collected, and all given gene details were converted into the National Center for Biotechnology Information (NCBI) official gene symbol by DAVID Bioinformatics Resource 2008. Entries unrecognized by DAVID Bioinformatics Resource 2008 were converted manually by searching the NCBI database. Whether the reported dysregulation was upregulation or downregulation was also noted for each gene. Candidate genes were selected by the following criteria: (1) reported in 3 or more microarray studies; (2) reported in 2 or more microarray studies with agreeing upregulation or downregulation that were not further confirmed; (3) all genes that have more than 10 results from Scopus search term “keloid” and “gene name” were excluded from the study; (4) include the genes that have been reported in any microarray and; (5) a list of genes from our own unpublished data (Table 3).

In addition, candidate genes were also selected from linkage regions reported by Marneros et al.^20^ Genes located between the markers D2S1328 and D2S2275 on chromosome 2 and between the markers D7S1818 and D7S4737 on chromosome 7 were identified using Ensembl (http://www.ensembl.org/index.html).

Thorough English language literature searches were carried out and genes likely to be involved in KD were selected. The selected genes were clustered through the tool, functional annotation clustering, provided in DAVID Bioinformatics Resources 2008 (http://david.abcc.ncifcrf.gov:8080/home.jsp). Annotation categories used for the functional clustering included all functional categories (COG_ONTOLOGY, PIR_SEQ_FEATURE, SP_COMMENT_TYPE, SP_PIR_ KEYWORDS, and UP_SEQ_FEATURE), all pathways (BBID, BIOCARTA, EC_NUMBER, KEGG_PATHWAY, and PANTHER_PATHWAY), all diseases (GENETIC_ASSOCIATION_DB_DISEASE, OMIM_DISEASE, and GENETIC_ ASSOCIATION_DB_DISEASE_CLASS), 3 protein domains (INTERPRO, PIR_ SUPERFAMILY, and SMART), and 3 gene oncology (GOTERM_BP_ALL, GOTERM_CC_ALL, and GOTERM_MF_ALL). The classification stringency was set to highest. Where possible, 1 candidate gene was selected from each cluster as a representative of the functional group.

### Selection for reference genes

The selection of suitable reference genes was carried out using GeNorm.^21^ Seven reference genes were used to screen for the most stably expressed reference genes, including β-actin (*ACT-β*), β-2-microglobulin (*β2M*), glyceraldehyde-3-phosphate dehydrogenase (*GAPDH*), hydroxymethyl-bilane synthase (*HMBS*), hypoxanthine phosphoribosyl-transferase I (*HPRT*), ribosomal protein L32 (*RPL32*), and succinate dehydrogenase complex subunit A (*SDHA*).^21^,^22^ The most stable reference genes across all biopsy, culture, normal, and margin samples were selected.

### Reverse transcriptase-quantitative polymerase chain reaction

Reverse transcriptase-quantitative polymerase chain reactions (RT-qPCRs) were carried out using LightCycler 480 platform (Roche Diagnostics GmbH). Polymerase chain reactions were performed in 384 multiwell plates (Roche Diagnostics GmbH). Three replicates of each reaction were carried out. The reaction volume was composed of 4 µL of 1:20 diluted template cDNA, 5 µL of LightCycler 480 Probes Master (Roche Diagnostics GmbH), 0.2 µM of each primer (Metabion International AG, Martinsried, Germany) (Table 4), 0.1 µL of probe from Universal Probe Library (Roche Diagnostics GmbH), and nuclease-free water (Ambion) to make up to a total volume of 10 µL. Four microliters of nuclease-free water was used to substitute for the template cDNA for the no template controls. LightCycler 480 software, Version 1.2 (Roche Diagnostics, Burgess Hill, England) was used to specify the qPCR conditions and to calculate the threshold cycle number (*C*_*T*_). The qPCR conditions were programmed as follows: First, there was 1 cycle at 95°C for 5 minutes for the activation of Hot Start Taq polymerase. Then, 45 amplification cycles were carried out; each cycle consisted of denaturation at 95°C for 10 seconds and annealing and extension at 60°C for 30 seconds. Finally, a cooling cycle was programmed at 40°C for 10 seconds. The reading of fluorescence level of the qPCRs in each amplification cycle was taken at the end of the 60°C step. The second derivative method was used for determining the *C*_*T*_.

### Gene expression–level analysis

The statistical significance of the difference in gene expression levels between the normal skin and margin was determined by the relative *C*_*T*_ method.^23^,^24^ The Δ *C*_*T*_ for each gene was obtained by deducting the average *C*_*T*_ for the reference transcripts from the *C*_*T*_ for the candidate transcripts (equation 1).^24^ Using a statistical method suggested by Yuan and Stewart,^24^ the statistical significance of the Δ *C*_*T*_ of each gene in the normal and margin samples was compared with each other by paired *t* test with the software SPSS, Version 14.0 (SPSS, Inc).

a (1) Candidate gene *C*_T_ – reference gene *C*_T_=Δ*C*_T_

Because 2 copies of amplicon should be obtained during each cycle of the PCR, 2^-Δ CT^ was used to represent the relative gene expression levels in natural numbers for presenting the results in bar charts (equation 2).

(2) 2^-Δ CT^ = Relative gene expression for the candidate gene

The average fold change between normal and margin samples was then calculated averaging the 2^-ΔΔ CT^ for all patients (equations 3 and 4).^23^

(3) Keloid margin Δ*C*_T_ – normal skin Δ *C*_T_=Δ Δ*C*_T_

(4) 2^-ΔΔ CT^ = Fold change of a candidate gene

**Figure 2:**
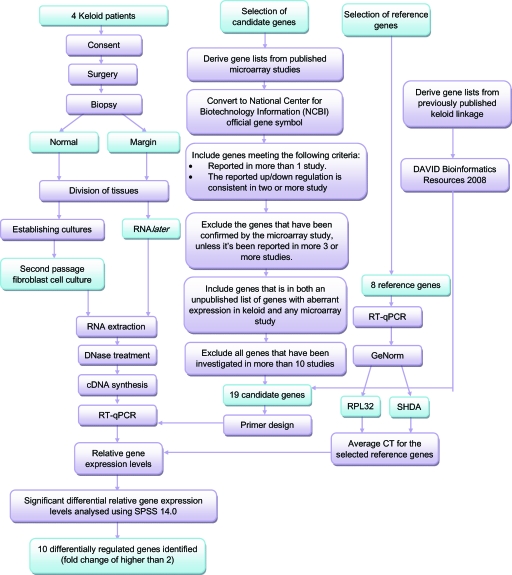
Flowchart summarizing steps taken and findings in this study. cDNA indicates complementary DNA; RPL32, ribosomal protein L32; RT-qPCR, reverse-transcription quantitative polymerase chain reaction; and SDHA, succinate dehydrogenase complex subunit A.

A summary of steps taken to identify the potential biomarkers for KD is shown in Figure 2.

## RESULTS

### Candidate gene selection

A total of 47 genes have been found to be present in 2 or more microarray studies. Fifteen candidate genes, including alpha-2-macroglobulin (*A2M*), aggrecan (*ACAN*), annexin A-1 (*ANXA1*), asporin (*ASPN*), chromosome 5 open reading frame 13 (*C5ORF13*), epidermal growth factor receptor (*EGFR*), hepatoma-derived growth factor (*HDGF*), hypoxia-inducible factor 1, alpha subunit (*HIF1A*), insulin-like growth factor binding protein 5 (*IGFBP5*), insulin-like growth factor binding protein 7 (*IGFBP7*), inhibin, beta A (*INHBA*), lectin, galactoside-binding, soluble, 1 (*LGALS1*), pleiotrophin (*PTN*), serpin peptidase inhibitor, clade F (*SERPINF1*), and serpin peptidase inhibitor, clade H (*SERPINH1*), were selected following the selection criteria.

**Figure 3:**
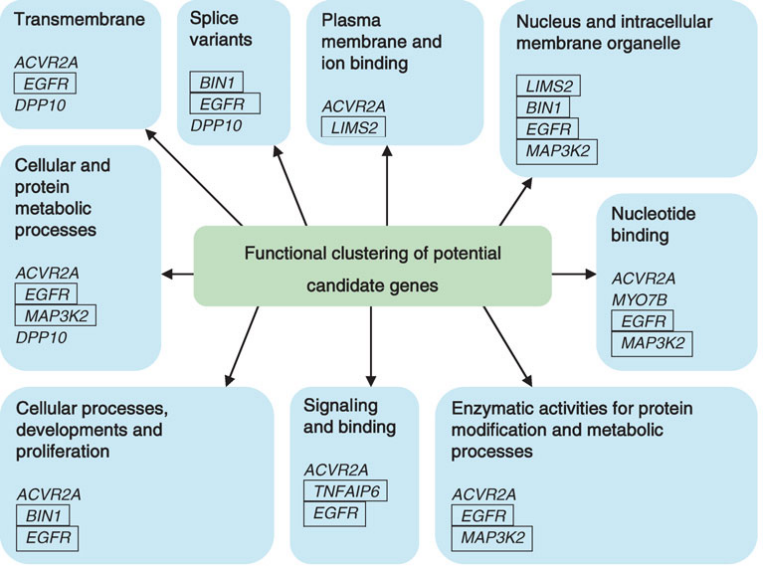
Functional clustering of the genes present within the keloid susceptibility loci. Selected genes present within keloid susceptibility loci, 7p11 and 2q23, have been functionally clustered using DAVID Bioinformatics Resources 2008 Functional Annotation Tool. The genes are separated into 9 separated category, and at least 1 gene from each categories has been selected for downstream quantitative polymerase chain reaction analysis. The selected genes are marked with a box around them. *EGFR* indicates epidermal growth factor receptor; *BIN1*, bridging integrator 1; *LIMS2*, LIM and senescent cell antigen-like domains 2; *MAP3K2*, mitogen-activated protein kinase kinase kinase 2; *TNFAIP6*, tumor necrosis factor-α inducible protein 6; *ACVR2A*, activin receptor IIA; *DPP10*, inactive dipeptidyl peptidase 10; and *MYO7B*, myosin VIIB.

One hundred and sixty-eight genes were identified to be present in the 2q23 keloid linkage (between markers D2S410 and D2S1353) and 50 genes in the 7p11 keloid linkage (between markers D7S1818 and D7S473). The list of candidate genes was shortlisted to 9 genes through a comprehensive literature search for genes that directly or indirectly contribute to keloid etiopathogenesis. The criteria included relations to cell proliferation and migration, fibrosis, inflammation, MAP kinase signaling, tissue homeostasis apoptosis, tumor progression, and wound healing. The 9 genes included tumor necrosis factor-α inducible protein 6 (*TNFAIP6*), activin receptor IIA (*ACVR2A*), mitogen-activated protein kinase kinase kinase 2 (*MAP3K2*), myosin VIIB (*MYO7B*), LIM and senescent cell antigen-like domains 2 (*LIMS2*), dipeptidyl peptidase 10 (*DPP10*), bridging integrator 1 (*BIN1*), epidermal growth factor receptor (*EGFR*), and von Willebrand factor C domain containing 2 (*VWC2*). Eight of the 9 input genes were clustered using DAVID Bioinformatics Resources 2008 into 9 groups according to their functional categories. Five genes were then selected (Fig 3), with at least 1 gene chosen from each cluster. The selected candidate genes included *TNFAIP6*, *LIMS2*, *MAP3K2*, *BIN1*, and *EGFR*.

### Reference gene selection

*RPL32* and *SHDA* were identified as most stably expressed reference genes across all samples. The pairwise variation in the 2 genes was less than 0.15, the threshold below which the inclusion of additional reference genes was not required. The 2 reference genes, *RPL32* and *SHDA*, were therefore used as the internal control genes for the normalization in RT-qPCRs.

### RNA quality

The values of RNA integrity number (RIN) for all samples were higher than 7.0; a RIN value of more than 5.0 indicated good total RNA quality, whereas a RIN value of more than 8.0 indicated best total RNA quality.^25^

### Gene expression levels of candidate genes

**Figure 4:**
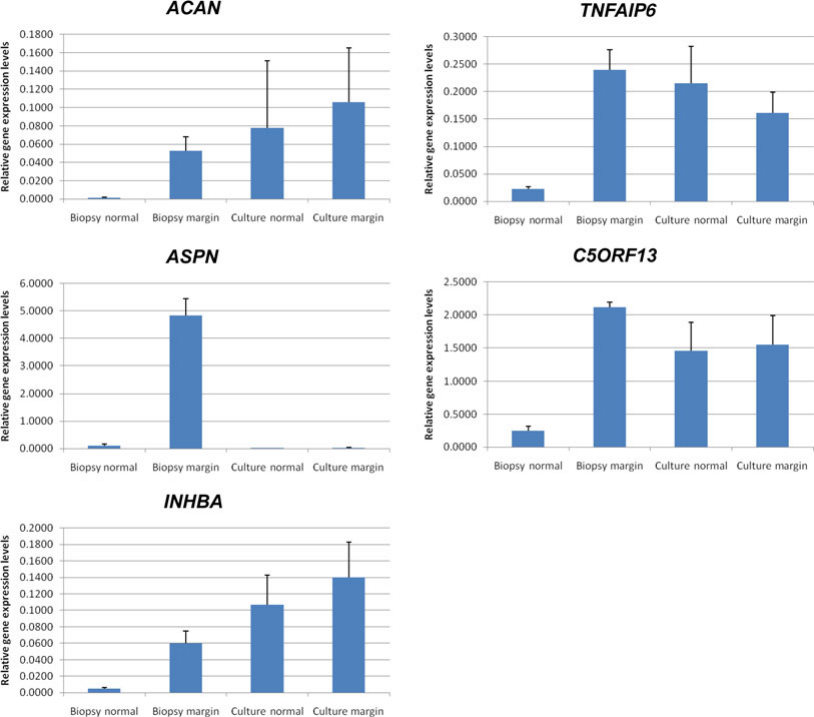
Relative gene expression levels in all samples for 5 genes that are highly upregulated in keloid margin. Significant upregulation have been observed in the following 5 genes in biopsies of keloid margin. However, this is not observed in the fibroblast culture equivalents. *ACAN* indicates aggrecan; *ASPN*, asporin; *INHBA*, inhibin, beta A; *TNFAIP6*, tumor necrosis factor-α inducible protein 6; and *C5orf13*, chromosome 5 open reading frame 13.

A statistically significant (*P* < .05) differential expression and a fold change of more than 2 were determined between keloid margin and internal control biopsy samples for 10 genes, including *ACAN*, *ASPN*, *C5orf13*, *HIF1A*, *IGFBP7*, *INHBA*, *LGALS1*, *PTN*, *SERPINH1*, and *TNFAIP6* (Table 5). *ACAN*, *ASPN*, *INHBA*, *TNFAIP6*, and *C5orf13* were the 5 candidate genes showing the highest average fold change in margin biopsies (fold change ≥ 9.9) (Fig 4). However, the significant high fold changes between normal and keloid margin samples was not observed in culture samples (Fig 4).

## DISCUSSION

Through comprehensive literature searches and bioinformatics analytic approaches, 15 candidate genes were identified from published microarray data sets and 4 from previously determined keloid linkage loci. Ten of these 19 genes, including *ACAN*, *ASPN*, *C5orf13*, *HIF1A*, *IGFBP7*, *INHBA*, *LGALS1*, *PTN*, *SERPINH1*, and *TNFAIP6* (Table 5), demonstrated a statistically significant difference for the gene expression levels between keloid margin and internal control skin.

The highest fold change between internal keloid normal and keloid margin was observed in ACAN, with a fold change of approximately 175 (*P* = .046). The presence and function of ACAN have been well characterized in cartilage but not in skin.^26^ ACAN interacts with hyaluronic acid, which has been demonstrated to be present at higher levels in keloid fibroblasts.^27^

ASPN also showed a high level of significant fold change (approximately 96-fold upregulation) in keloid margins. Similar to ACAN, it is also a protein found in cartilage. ASPN polymorphism and abundance have been associated with osteoarthritis, a disease characterized by progressive cartilage degeneration.^28^ A 96-fold upregulation of ASPN has been observed in keloid margin (*P* = .004). ASPN has been shown to directly bind to transforming growth factor (TGF)-β_1_ in vitro and is suggested to be a negative regulator for TGF-β in cartilage.^28^,^29^

Similarly, C5ORF13, also known as P311, has also been implicated to be a negative regulator for TGF-β_1_.^30^ The expression of C5ORF13 is shown to be upregulated by approximately 10-fold in keloid margin (*P* = .000). The expression of C5ORF13 has been shown to induce nonfibrogenic myofibroblast-like phenotype in 3T3 cells, including upregulation of smooth muscle α-actin, basic fibroblast growth factor, vascular endothelial growth factor, platelet-derived growth factor (PDGF), PDGF receptors and integrins α_3_ and α_5_.^30^ However, unlike what is normally found in typical myofibroblasts, P311-induced myofibroblasts downregulate TGF-β_1_ and its receptor TGF-βR2.^30^ The authors have also suggested that C5ORF13 reduces the expression of matrix metalloproteinase (MMP)-2 and MMP-9 mRNA.^30^ It has been postulated that the expression of C5ORF13 is found in human wound myofibroblast precursors and myofibroblasts; C5ORF13-induced myofibroblasts are thought to migrate in an ameboid pattern on fibrin structures found in the initial wound matrix, and the ameboid pattern is reversed to mesenchymal pattern upon stimulation with TGF-β_1_.^31^ Furthermore reduced degradation of fibrin has been observed in keloid.^32^ and it is possible this ameboid migration pattern contributes to the aggressive characteristics of the keloid margin.

INHBA showed a significant 14-fold upregulation in keloid margin samples (*P* = .031). Previously *INHBA* has been suggested to be a possible target gene by Seifert et al^7^ in a microarray study performed on fibroblasts derived from keloids and external control skin. The authors observed an increased mRNA expression of INHBA in fibroblasts derived from all lesional sites of keloids when compared with external control skin. However, a downregulation of INHBA protein level was observed when comparing keloid margin with external normal skin.^7^ In addition, Seifert et al^7^ also noted an increased expression of inhibitor for INHBA at the margin. The significant difference in gene expression levels that Seifert et al observed between keloid and normal fibroblasts was observed only in RNA samples extracted from biopsies, not the fibroblast cultures, in this study. This difference may have been a result of the culturing conditions, which will be discussed in detail.

Smith et al^12^ determined significant downregulation of PTN in keloid fibroblast cultures by microarray analysis. However, in our study, an approximate 8-fold significant upregulation (*P* = .041) was observed in keloid biopsies for PTN, which correlates to PTN upregulation reported in keloid biopsies by Chen et al^17^ and Hu et al^13^ by microarray analysis. The discrepancy between the observations may be due to the different RNA sources (e.g., from fibroblast cultures or biopsy tissues).

The overexpression of SERPINH1, which showed a 5.7-fold upregulation in keloid margin in this study (*P* = .003), has been suggested to promote excessive collagen deposition in keloids.^33^ LGALS1 is a type of lectin that has been implicated in cell-cell and cell-matrix interactions and has been suggested to be involved in tumor progression, at least partly, through the induction of T-cell apoptosis.^34^ IGFBP7 shows a 2.8-fold upregulation in keloid margins (*P* = .022). Several other insulin-like growth factor–binding proteins have been demonstrated to be differentially expressed in keloids.^35^ HIF-1α shows a 2.1-fold upregulation in keloid margins investigated in this study (*P* = .006). The expression of aberrent HIF-1α and plasminogen activator inhibitor-1 (PAI-1), a downstream molecule affected by HIF-1, in keloids has been well documented.^36^ In addition, treatment against HIF-1α leads to downregulation of PAI-1.^37^

A statistically significant (*P* = .002) finding was observed for the higher level of TNFAIP6 transcripts in keloid margins. TNFAIP6 was shown to be involved in the inhibition of neutrophil migration, modulation of inflammation, and tissue remodeling.^38^ In individuals with renal fibrosis, increased expression of TNFAIP6 was reported in the proximal tubular epithelial cells, a cell population that was demonstrated to have the potential to contribute to the pathogenesis of renal fibrosis.^39^ Similar to ACAN, TNFAIP6 also interacts with hyaluronic acid, which has been shown to be upregulated in keloid fibroblasts.^27^ Although Marneros et al^20^ did not identify mutations or disease-associated polymorphisms in the *TNFAIP6* gene when screening genomic DNA of the affected and unaffected family members of the Japanese family used to establish the 2q23 keloid linkage. There may however be other undetected chromosomal abnormalities, such as mutations within introns.

While 10 of the investigated genes demonstrated significant differential gene expression between the biopsies of keloid margin and internal control, no significant differences in gene expression levels were observed for any genes between their fibroblast culture equivalents. This observation suggested that careful interpretation must be done for the transcriptomic analysis obtained from tissue cultures. Similarly, Dangles et al^40^ demonstrated that culturing conditions have a profound impact on gene expression of bladder cancers. On the other hand, Bignotti et al^41^ suggested that the use of short-term culturing (passage 0) could enhance the purity of the ovarian serous papillary carcinoma (OSPC) tumor cell population and without altering the *OSPC* gene expression patterns. The inconsistency may be due to the following reasons: different genes of interest for different disease, different experimental methods (such as the techniques used for the gene expression studies and culturing conditions) and the use of different passages.

In this study, a significant upregulation was observed for 10 of the 19 studied transcripts in the biopsy samples of keloid margin when compared with normal skin adjacent to keloid lesions (*P* < .05). These identified genes, including *ACAN*, *ASPN*, *C5ORF13*, *EGFR*, *HDGF*, *HIF1A*, *IGFBP7*, *INHBA*, *LGALS1*, *PTN*, *SERPINH1*, and *TNFAIP6*, may serve as potentially important biomarkers for KD.

## Acknowledgments

The authors thank the support of the following organization for this study: NIHR (UK). In addition Miss H Shah's contribution to the Bioinformatics search is also acknowledged. The authors specially extend their gratitude to the GAT family Foundation, Steve and Kathy Fitzpatrick, and the North American Foundation of the University of Manchester.

## Figures and Tables

**Table 1:** Patient details

Study ID	Age, y	Gender	Race/ethnicity	Site
KS41	34	Female	White	Shoulder
KS42	24	Female	White/black Caribbean	Sternum
KS43	30	Female	White	Shoulder
KS44	26	Female	White	Sternum

**Table 2:** Microarray study details

Author	Year	Number of targets	Culture/biopsy	Control	Number of reported dysregulated targets	Number of given dysregulated gene target	Number of targets converted to official gene symbol
Smith et al^12^	2008	Affymetrix GeneChip (U133 Plus 2.0 Array)	Culture	External control	904	583	577
Seifert et al^7^	2008	Affymetrix GeneChip (U133 Plus 2.0 Array)	Culture	External control	578 (bottom) 599 (top) 632 (margin)	46	46
Satish et al^15^	2006	Affymetrix GeneChip (U133A)	Culture	External control	48	48	32
Leppert et al^18^	2006	No normal control					
Hu et al^13^	2006	8064 (gene; CSC-GE-80)	Biopsy	External control	277	259	259
Naitoh et al^14^	2005	9128 (targets)	Culture	External control	32	32	31
Na et al^19^	2004	3063 (targets)	Biopsy	Internal control	9	9	9
Chen et al^16^	2004	Replicate of Chen et al (2003)					
Chen et al^17^	2003	8464 (targets)	Biopsy	Internal control	402	99	54

**Table 3:** Genes selected from microarray studies

Official gene symbol	Number of microarray reports	Studies mentioned	Reported upregulation/downregulation	Biopsy or culture
*A2M*	2	Hu et al (2006)	Up	Biopsy
		Chen et al (2003)	Up	Biopsy
*ACAN*	2	Seifert et al (2008)	Up	Culture
		Naitoh et al (2005)	Up	Culture
*ANXA1*	2	Hu et al (2006)	Up	Biopsy
		Seifert et al (2008)	Up	Culture
*ASPN*	1	Smith et al (2008)	Up	Culture
*C5ORF13*	3	Naitoh et al (2005)	Up	Culture
		Hu et al (2006)	Up	Biopsy
		Smith et al (2008)	Up	Culture
*EGFR*	2	Hu et al (2006)	Down	Biopsy
		Seifert et al (2008)	Down	Culture
*HDGF*	2	Hu et al (2006)	Down	Biopsy
		Chen et al (2003)	Down	Biopsy
*HIF1A*	2	Hu et al (2006)	Up	Biopsy
		Seifert et al (2008)	Up	Culture
*IGFBP5*	3	Hu et al (2006)	Down	Biopsy
		Seifert et al (2008)	Up	Culture
		Smith et al (2008)	Up	Culture
*IGFBP7*	2	Hu et al (2006)	Up	Biopsy
		Seifert et al (2008)	Up	Culture
		Smith et al (2008)	Down	Culture
*INHBA*	1	Seifert et al (2008)	Up	Culture
*LGALS1*	1	Hu et al (2006)	Up	Biopsy
*PTN*	3	Chen et al (2003)	Up	Biopsy
		Hu et al (2006)	Up	Biopsy
		Smith et al (2008)	Down	Culture
*SERPINF1*	2	Hu et al (2006)	Down	Biopsy
		Na et al (2004)	Down	Biopsy
*SERPINH1*	2	Hu et al (2006)	Up	Biopsy
		Smith et al (2008)	Up	Culture

A2M indicates alpha-2-macroglobulin; ACAN, aggrecan; ANXA1, annexin A-1; ASPN, aspirin; C5ORF13, chromosome 5 open reading frame 13; EGFR, epidermal growth factor receptor; HDGF, hepatoma-derived growth factor; HIF1A, hypoxiainducible factor 1, alpha subunit; IGFBP, insulin-like growth factor binding protein; INHBA, inhibin, beta; LGALS1, lectin, galactoside-binding, soluble, 1; PTN, pleiotrophin; SERPINF1, serpin peptidase inhibitor, clade F; and SERPINH1, serpin peptidase inhibitor, clade H.

**Table 4:** Primer details

Official gene symbol	Transcript ID	Forward primer	Reverse primer	Probe sequence
*A2M*	NM_000014.4	ctacgagacggatgagtttgc	cttctgtggagctctgagaaca	tgctggag
*ACAN*	NM_001135.2	cagatggacaccccatgc	cttctgtggagctctgagaaca	catcacca
	NM_013227.2			
*ANXA1*	NM_000700.1	aatgcacagcgtcaacagat	tgtttcatccaggggcttt	tctccagg
*ASPN*	NM_017680.3	gacaccatgaaggagtatgtgc	aagaagggtttggcagagc	ttcctggc
*C5orf13*	NM_004772.1	tgtcccaaaggaagtgaacc	attcttggggagcggagtt	tgggcagc
*EGFR*	NM_201284.1	catgtcgatggacttccaga	gggacagcttggatcacact	tgggcagc
	NM_005228.3			
	NM_201282.1			
	NM_201283.1			
*HDGF*	NM_004494.2	acgagaaaggagcgttgaag	tccttgggacgtttaggaga	tgctggag
	NM_001126050.1			
	NM_001126051.1			
*HIF1A*	NM_181054.1	cagctatttgcgtgtgagga	cagctatttgcgtgtgagga	ggatgctg
	NM_001530.2			
*IGFBP5*	NM_000599.3	aagcagggaacgcatgatt	aagcagggaacgcatgatt	ccaggctg
*IGFBP7*	NM_001553.1	ctgtcctcatctggaacaagg	tgaatggccaggttgtcc	ggcaggag
*INHBA*	NM_002192.2	ctcggagatcatcacgtttg	ccttggaaatctcgaagtgc	gccaggaa
*LGALS1*	NM_002305.3	catcgtgtgcaacagcaag	acacctctgcaacacttcca	ggaggctg
*PTN*	NM_002825.5	aactgaccaagcccaaacct	ggtgacatcttttaatccagca	caggagaa
*SERPINF1*	NM_002615.4	acgctatggcttggattcag	atactcatgcttccggtcaag	ttgcccag
*SERPINH1*	NM_001235.2	tgatgatgcaccggacag	gatggggcatgaggatgat	ggctggag
*TNFAIP6*	NM_007115.2	ggccatctcgcaacttaca	cagcacagacatgaaatccaa	agaggcag
*MAP3K2*	NM_006609.3	aaggctatggaagaaaagcaga	tggctgagtggcgatttta	attgctgc
*LIMS2*	NM_017980.2	ctgcgagactcactacaacca	gacaccacatcgccttcaat	gcagccat
*BIN1*	NM_139344.1	gtgatccccttccagaacc	ttccagtcgctctccttcac	tcctgctc
	NM_139345.1			
	NM_139346.1			
	NM_139348.1			
	NM_139347.1			
	NM_139349.1			
	NM_139351.1			
	NM_139350.1			
	NM_139343.1			
	NM_004305.2			

A2M indicates alpha-2-macroglobulin; ACAN, aggrecan; ANXA1, annexin A-1; ASPN, aspirin; BIN1, bridging integrator 1; C5ORF13, chromosome 5 open reading frame 13; EGFR, epidermal growth factor receptor; HDGF, hepatoma-derived growth factor; HIF1A, hypoxiainducible factor 1, alpha subunit; IGFBP, insulin-like growth factor binding protein; INHBA, inhibin, beta; LGALS1, lectin, galactoside-binding, soluble, 1; LIMS2, LIM and senescent cell antigen-like domains 2; MAP3K2, mitogen-activated protein kinase kinase kinase 2; PTN, pleiotrophin; SERPINF1, serpin peptidase inhibitor, clade F; SERPINH1, serpin peptidase inhibitor, clade H; and TNFAIP6, tumor necrosis factor, alpha-induced protein 6.

**Table 5:** Significant upregulation/downregulation

		Probability	Average fold change
Official gene symbol	Gene name	Biopsy	Culture	Biopsy	Culture
*A2M*	alpha-2-macroglobulin	0.109	0.292	2.4	2.7
*ACAN*	aggrecan	0.046	0.676	174.7	20.5
*ANXA1*	annexin A-1	0.061	0.120	1.2	0.9
*ASPN*	asporin	0.004	0.822	96.3	3.2
*C5ORF13*	chromosome 5 open reading frame 13	0.000	0.868	9.9	1.4
*EGFR*	epidermal growth factor receptor	0.003	0.185	0.6	0.7
*HDGF*	hepatoma-derived growth factor	0.004	0.827	0.7	1.1
*HIF1A*	hypoxia-inducible factor 1, alpha subunit	0.006	0.265	2.1	0.8
*IGFBP5*	insulin-like growth factor binding protein 5	0.304	0.665	1.8	0.9
*IGFBP7*	insulin-like growth factor binding protein 7	0.022	0.226	2.8	1.5
*INHBA*	inhibin, beta A	0.031	0.636	14.0	3.0
*LGALS1*	lectin, galactoside-binding, soluble, 1	0.002	0.238	5.1	1.2
*PTN*	pleiotrophin	0.041	0.912	7.8	0.9
*SERPINF1*	serpin peptidase inhibitor, clade F	0.115	0.649	2.6	1.0
*SERPINH1*	serpin peptidase inhibitor, clade H	0.003	0.390	5.7	1.2
*TNFAIP6*	tumor necrosis factor-α inducible protein 6	0.002	0.559	11.4	1.0
*MAP3K2*	mitogen-activated protein kinase kinase kinase 2	0.006	0.417	1.3	0.9
*LIMS2*	LIM and senescent cell antigen-like domains 2	0.580	0.582	0.9	1.5
*BIN1*	bridging integrator 1	0.248	0.919	1.6	1.1
